# Molecular Analysis of Echovirus 13 Isolates and Aseptic Meningitis, Spain

**DOI:** 10.3201/eid0908.030080

**Published:** 2003-08

**Authors:** Ana Avellón, Inmaculada Casas, Gloria Trallero, Carmen Pérez, Antonio Tenorio, Gustavo Palacios

**Affiliations:** * Instituto de Salud Carlos III, Madrid, Spain; † Doctor Negrín Hospital, Las Palmas de Gran Canaria, Canary Island; ‡ Columbia University, New York, New York, USA

**Keywords:** Enterovirus, human, echovirus, Echovirus 13, Meningitis, Viral, Meningitis, Aseptic, Molecular Epidemiology, Spain, Canary Islands , research

## Abstract

Echovirus 13 (EV13), considered rare, was reported worldwide in 2000, mostly related to aseptic meningitis outbreaks. In Spain, 135 EV13 isolates were identified. The genetic relationships between 64 representative strains from Spain and other reported isolates from the United States, Germany, Italy, Japan, and Sweden were described by analyzing the partial sequence of the major capsid protein (VP1) gene. The strains from Spain were clearly identified as EV13 (79.5% similarity with the EV13 reference strain) and were grouped phylogenetically into two different clusters (by origination on either the Iberian Peninsula or Canary Islands). Isolates from Germany from 2000 clustered with the Canary Islands group. The isolates from other countries obtained before 2000 were genetically distant. Changes in EV13 coding sequence involved several differences in the C-terminal extreme of the VP1 protein. Part of the neutralizing antigenic site III has been described in this genome region in poliovirus and swine vesicular disease virus.

The human enteroviruses (HEVs; *Enterovirus* [ENV] genus, *Picornaviridae* family) are common human pathogens with a worldwide distribution. HEVs are found in temperate climates and especially in the tropics because warm weather favors their spread. Humans are the only known reservoir for HEV, and the main transmission route is fecal-oral. HEV infections may be unapparent or related to different disorders, including encephalitis, pleurodynia, myocarditis, conjunctivitis, or systemic infections in neonates. These viruses are commonly reported in association with aseptic meningitis outbreaks in pediatric patients (*1*). Although five HEV species exist (*2*), they are grouped into four clusters (HEV-A, -B, -C, and -D) on the basis of sequence analysis of the major capsid protein (VP1) and the 3′ noncoding region (*3*). The HEV-B cluster includes coxsackie virus B (CBV), coxsackie virus A9, ENV69, and all echoviruses (EV).

The advent of nucleic acid amplification methods has facilitated the study of the molecular epidemiology of HEV (*4*–*6*). Several reverse transcription-polymerase chain reaction (RT-PCR) methods were developed for this purpose, most of which analyze different sequence fragments within the VP1 gene (*7*–*9*). Moreover, the amplification and subsequent analysis of the VP1 3′ end partial gene have been successfully used to serotype HEVs (*10*,*11*) and to describe the molecular epidemiology of EV30 (*12*). Analysis of this fragment of the enteroviral genome has permitted the study of important pathogenic structures, such as part of the antigenic sites described for polioviruses (*13*), CBV, and swine vesicular disease virus (SVDV) (*14*,*15*), and part of the canyon structure, related to viral attachment, which has been described in several HEVs and rhinoviruses (*16*).

The EV strains most frequently isolated in patients with aseptic meningitis are EV30, EV6, EV11, and EV9 (*1*). Displacement of prevailing lineages on the basis of immune escape (antigenic drift) has been suggested for EV30 as the mechanism to maintain viral circulation levels that increase periodically (*12*,*17*,*18*). Unlike EV30, EV13 has been considered a rare virus (*1*). Until 1999, isolation data showed very low rates in countries such as the United States (*19*), Ireland (*20*), and England and Wales (*21*). During 2000, EV13 circulation, mostly related to aseptic meningitis, was reported in America (*19*), Europe (*20*,*22*–*25*), Asia (*26*), and Australia (*27*). In Spain, an outbreak of aseptic meningitis attributable to EV13 occurred from February to October 2000 (*25*), the first identification of this virus in Spain since record keeping began in 1988 (*28*).

We describe the molecular epidemiology of the EV13 isolates obtained in Spain from the 2000 outbreak by analyzing the VP1 partial sequences of 64 identified viruses. We also compare them with other isolates for which sequences have been deposited in databases. Finally, by analyzing the molecular nature of their changes in nucleotides and amino acids, we attempt to describe the nature of, and reasons for, the actions and epidemiology of EV13.

## Materials and Methods

### Clinical Specimens

**Figure 1:**
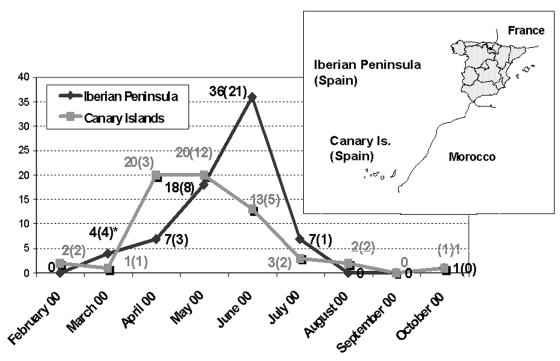
Temporal distribution of the Spanish echovirus 13 isolates during 2000. The isolates were grouped attending to their geographic origin as from the Iberian Peninsula and from the Canary Islands. The number of viruses included in the study (in parentheses) as well as the total isolates per month are shown. *Includes three sequences obtained directly from cerebrospinal fluid.

Study specimens consisted of 61 EV13 isolates and three cerebrospinal fluid (CSF) samples obtained as follows. During 2000, the Enterovirus Laboratory (Service of Virology, National Microbiology Centre, Madrid) received 538 isolates for typing from different laboratories within Spain. These viruses were mostly recovered in a human rhabdomyosarcoma cell line with a positive cytopathic effect, which was confirmed by immunostaining with an anti-HEV group-specific antibody (Dako, Glostrup, Denmark). A neutralization test (Lim-Benyesh-Melnick immune serum pools) identified EV13 in 135 (25%) specimens. On the basis of their geographic and temporal distribution (Figure 1), 61 isolates were selected for further study. The selected isolates were obtained from CSF (n=44), stool (n=12), and pharyngeal swab (n=5) samples from patients with aseptic meningitis, except for samples from seven patients who had fever, one who had acute flaccid paralysis, and two healthy persons (contacts of the patients with acute flaccid paralysis). The outbreak was carefully studied in the Canary Islands because of the high rate of infected patients and the context in which the cases occurred, including geographic location, climate, and tourism activity in the area during the year. Three additional CSF samples obtained from patients with aseptic meningitis in March 2000 were supplied by the Microbiology Diagnostic Service (National Microbiology Centre, Madrid, Spain).

### Amplification

The VP1 3′ end genome region, successfully used to study the molecular epidemiology of other HEVs (*7*,*12*), was amplified in all 64 isolates. A 609-nucleotide (nt) fragment was amplified by using the RT-nested PCR method (*10*) with a total of 5 μL of each 1:10 diluted cell culture isolate. A fragment of the expected length was also obtained in the three additional CSF samples, previously extracted with guanidinium thiocyanate lysis buffer (*29*).

The VP1 3′ end fragment was useful in differentiating and studying the epidemiology of HEVs. However, since our main goal was to compare the sequences of the Spanish isolates with those of isolates from the rest of the world, we designed a specific PCR to amplify the VP1 5′ end gene, where a collection of isolates from Germany had been sequenced (*22*). For that purpose, specific primers of EV13 were designed by using the multiple alignments constructed with the available EV13 sequences employing Clustal X software (free software available from: URL: http://www-igbmc.u-strasbg.fr/BioInfo/)**.** The primer sequences were as follows: EV13_VP1sense (5′–3′) TGAGACAGGGCACACATC; EV13_VP1anti (5′–3′) GCTAATGAATGGGATGGACAT. Its relative positions to EV11 polyprotein genome (X80059) were 2560–2577 and 2999–3019, respectively. Single-step RT and amplification was performed by using the Access RT-PCR kit (Promega Corp., Madison, WI). Five microliters of selected 1:10 diluted cell culture isolates was added to the reaction mixture which contained the following: 10 μL of 5X reaction buffer; 2 mM magnesium sulfate; 250 μM each of dATP, dCTP, dGTP, and dTTP; 0.4 μM of each EV13_VP1sense and EV13_VP1 anti-primers; 1 U of avian myeloblastosis virus RT; and 1 U of Thermus flavus DNA polymerase and Rnase-free distilled water to a final volume of 50 μL. Amplification was performed in a PTC-200 Peltier thermal cycler (M.J. Research, Inc., Waltham, MA), programmed for a first RT step of 45 min at 48°C, followed by 2 min at 94°C, and for 45 cycles of 30 sec for denaturation at 94°C, 2 min for annealing at 60°C, and 30 sec for elongation at 68°C. Elongation was extended for 5 additional minutes in the last cycle. PCR products were detected by electrophoresis on 2% agarose gels stained with ethidium bromide. A 460-nt fragment was amplified in 14 representative selected Spanish isolates.

### Sequencing and Sequence Analysis

Cycle sequencing reactions of products from both RT-PCR assays were performed by using the Big Dye terminator kit (Applied Biosystems, Foster City, CA). Ambiguities were resolved by sequencing both sense and antisense strands.

The 423-nt VP1 3′ end fragment (nt 2912–3334, according to the EV11 polyprotein genome, X80059) was analyzed through the multiple sequence comparison of the 64 viral sequences from Spain, the EV13 and ENV69 prototype strains (Del Carmen and Toluca-1, respectively), and the isolates VA86-6776 (*30*) and TX95-2089 (*7*). For the analysis of the 279-nt VP1 5′end fragment (nt 2609–2887, according to the EV11 polyprotein genome, X80059), sequences available from GenBank of the following isolates were included: 12 isolates from Germany, obtained during 1965, 1974, 1976, 1979, 2000, and 2001; one isolate each from Italy (1996) and Sweden (1999), and two from Japan (2001). This second group of sequences was compared with the corresponding VP1 5′ end gene of 14 Spanish representative isolates selected according to their geographic origin (four from the Canary Islands and within the Iberian Peninsula, four from the north, four from the center of the peninsula, and two from the east).

Multiple sequence alignments were built with Clustal X. For the molecular typing, Spanish VP1 3′ end gene sequences were compared pairwise with the HEV prototype strains to obtain the identity score (*11*). The percentage of similarity of the isolates with respect to the reference strain was calculated through Megalign software (DNASTAR, Madison, WI).

**Figure 2:**
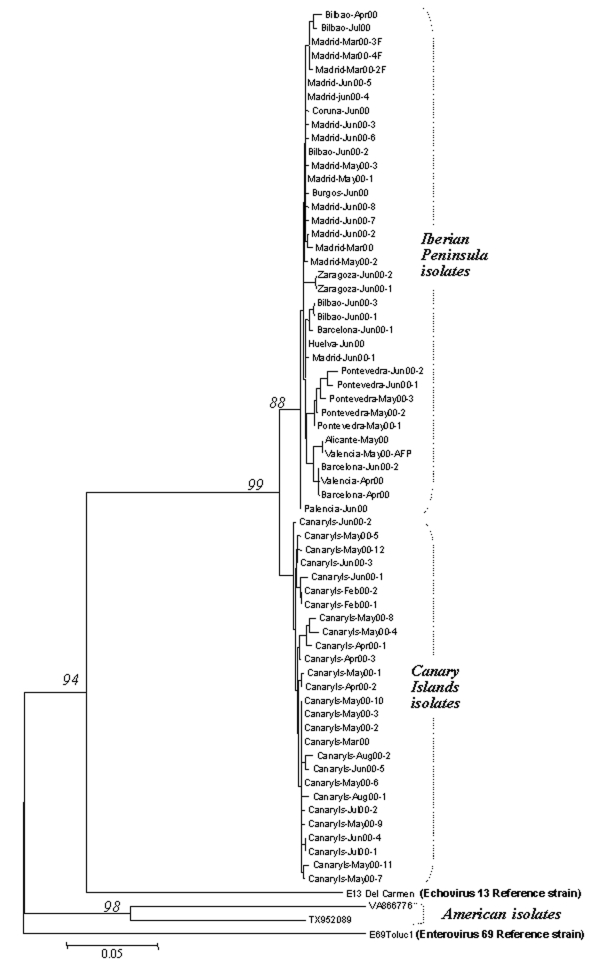
Phylogenetic tree of the VP1 3′ terminal region, which identifies the Spanish isolates as echovirus 13 (EV13) and differentiates two clusters (Iberian Peninsula and Canary Islands). The sequences included are, apart from the reference strains, all the Spanish EV13 (61 isolates and 3 sequences obtained directly from cerebrospinal fluid) and two American isolates. Model of nucleotide substitution: Kimura two parameters. Phylogenetic tree reconstructed with the neighbor joining method, and bootstrap analysis with 1,000 pseudoreplicate datasets.

**Figure 3:**
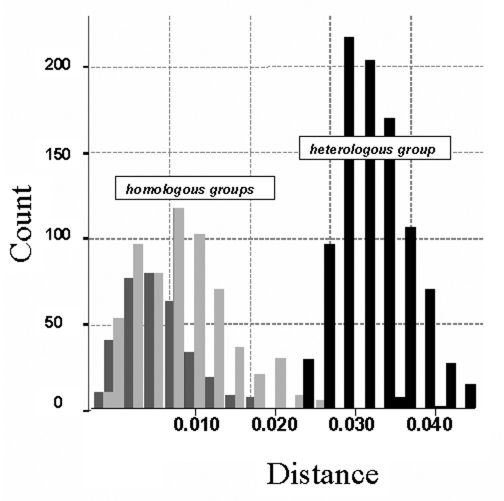
Histogram of the observed distances (Kimura two parameters method) within Canary Islands and Iberian Peninsula groups (dark gray) and between them (light gray). Analysis of variance (ANOVA) (one-way) test results: F=5238 (p=0.000). Distances within homologous groups: Canary Islands (light gray): mean 0.008 (standard deviation [SD] 0.004; n=351); Iberian Peninsula (dark gray): mean 0.010 (SD0.006; n=666). Distances between heterologous groups (black): mean 0.033 (SD 0.005; n=999).

**Figure 4:**
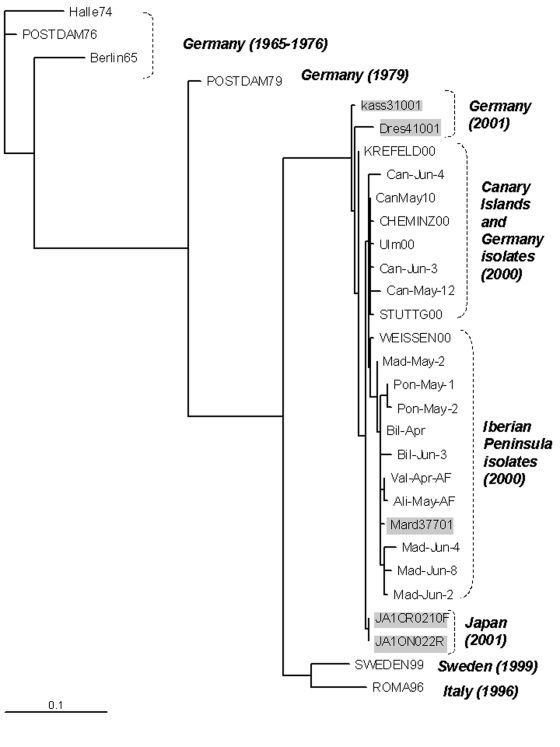
Phylogenetic tree of the 5′ VP1 extreme showing the relationship between the Spanish isolates and others. The sequences included are the 14 Spanish echovirus 13 selected isolates as well as the German (n=12), Italian (n=1), Swedish (n=1), and Japanese (n=2) ones. Phylogenetic tree reconstructed with the maximum-likelihood method, 50 Jumble. Gray sequences correspond to the 2001 isolates.

The phylogenetic tree in Figure 2 was reconstructed through the neighbor-joining method (MEGA version 2.1 software package; available from URL: http://www.magasoftware.net) by using Kimura two parameters as substitution model, with statistical significance of phylogenies estimated by bootstrap analysis with 1,000 pseudoreplicate datasets. The transition-transversion ratio employed (TTRatio=2) was estimated through the Tree-puzzle version 5.0 software (available from: URL: http://www.tree-puzzle.de)**.** Genetic distances were calculated using the same model of nucleotide substitution. The distance matrix was recorded and the pairwise observed distance values were employed to define different subgroups and to build the histogram in Figure 3.. The analysis of variance one-way test, with a statistical significance value of 0.05, was used to compare the means of the pairwise observed distance value matrix. To reconstruct the phylogenetic tree shown in Figure 4, the maximum-likelihood method (DNAML program, PHYLIP software [available from: URL: http://evolution.genetics.washington.edu]), was used, with hidden Markov as the model of nt substitution. A Poisson correction model was used to compare the amino acid sequences.

### Three-Dimensional Structure

The VP1 amino acid changes of the Spanish isolates with respect to the EV13 reference strain were projected onto the three-dimensional structure of EV11 (GenBank 1H8TA) (*31*) by using the program Cn3D (available from: URL: http://www.ncbi.nlm.nih.gov/Structure). Neutralizing antigenic sites previously reported for poliovirus (*13*), CBV4 (*32*), and CBV5 and SVDV (*14*,*15*) were localized in the structure. Amino acids were numbered according to EV11 sequence.

### Nucleotide Sequence Accession Numbers

Fourteen complete gene sequences of Spanish EV13 VP1 were submitted to the GenBank database under accession numbers AY227334–AY227347. Fifty Spanish EV13 VP1 3′ end fragments were also submitted under accession numbers AY227284–AY227333. These EV13 sequences were also included in this study: AF081327 (Del Carmen), AJ309256 (Roma96), AF401360 (Halle74), AF401359 (Postdam76), AF401358 (Postdam79), AF401357 (Berlin65), AF401356 (Chemniz00), AF401355 (Krefeld00), AF401354 (Stuttgart00), AF401353 (Ulm00), AY007223 (Weissenfels00), AF295467 (Sweden99), AF152299 (VA86-6776), AF081635 (TX95-2089), AB092985 (JA1CR0210F), AB092984 (JA1ON022R), AY131288 (KASS31001), AF538285 (DRES41001), and AF538284 (MARD37701). The sequence AF081349, corresponding to the ENV69 strain Toluca-1, was also included as the nearest possible taxon and outgroup. GenBank accession numbers of the rest of the VP1 partial gene of HEVs prototype strains have been detailed previously (*7*).

## Results

### Molecular Typing of Isolates

A preliminary multiple sequence alignment, as described by Palacios et al. (*11*), grouped all the Spanish viruses with the EV13 reference strain with 79.5% identity (SD [standard deviation] 0.37). ENV69 reference strain was the nearest taxon with 74.3% (SD 0.33) identity; the rest of the HEVs shared <71%. In addition, pairwise sequence distance comparison showed that all the isolates from Spain differed from the EV13 and ENV69 reference strains with observed nucleotide distances of 0.260 (standard error [SE] 0.028) and 0.344 (SE 0.032), respectively. Consequently, all the Spanish isolates were classified as EV13, in agreement with the neutralization test results.

### Comparison of the Nucleic Acid of the Spanish Sequences

Spanish isolates grouped into two major clusters corresponding to their respective geographic origin (Iberian Peninsula and Canary Islands) with statistical significance (Figures 2 and 3). The 10-nt changes that determined the groups were all in third codon position. The nucleotide distances observed within the Canary Islands sequence group ranged from 0 to 0.022 nt, whereas within the Iberian Peninsula group, the range was from 0 to 0.029. The distance range obtained after comparison of sequences belonging to these different groups was 0.024–0.052. The study of the temporal pattern of the Spanish outbreak suggests that, although all the Spanish isolates have a common source, the group from the Canary Islands might be closer to the ancestor.

### Comparison of the Spanish Isolates with Other Available EV13 Sequences

EV13 viruses isolated in 2000 and 2001 from Germany, Japan, and Spain were phylogenetically close. Within all of the studied sequences, the more similar ones, with a nucleotide distance of 0.011, corresponded to the strains isolated in Germany and the Canary Islands in 2000. The study of the temporal pattern by the maximum-likelihood approach also grouped the German 2000 isolates with the Spanish ones (Figure 4).

For the older European strains, obtained before 2000, the isolates were genetically more distant from the Spanish isolates; the 1999 strain from Sweden was the second most similar to the Spanish isolates, followed by the isolate from Italy (1996) and those from Germany (1979, 1976, and 1965). The two strains from the United States (VA86-6776 and TX95-2089) were the most distant from the Spanish isolates, with a genetic distance of 0.345 and 0.311, respectively (Figure 2). These two viruses seemed to be as similar to ENV69 as to EV13. Further sequence analysis of other strains of American EV13 and additional ENV69 isolates could clarify this point. However, although no other ENV69 sequences were available for comparison, we could confirm that these sequences did not cluster into the same evolutionary lineage of all other published EV13.

### Comparison of Amino Acid Sequences

**Figure 5:**
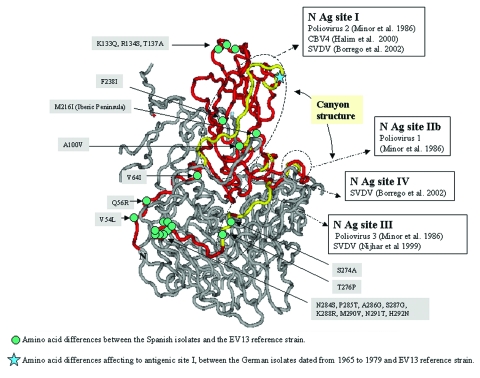
Mapping of the echovirus (EV) 13 VP1 amino acids. The three-dimensional structure is projected onto EV11 structure (GenBank accession no. 1H8TA) with VP1 amino acid numbering according to EV11. The sequenced fragment is shown in red. Previously reported neutralizing antigenic sites for poliovirus, CBV4, and SVDV are shown in yellow. The enterovirus canyon structure is found in a similar location to the canyon structure of poliovirus as reported by (*40*).

Several amino acid differences could be observed between the Spanish isolates and the EV13 reference strain. The EV13 neutralization sites have not been determined. However, they have been reported for other ENVs and some of them (sites I and III) are in the same position and have the same amino acids involved. For this reason, the amino acid changes noticed in this study and neutralization sites previously reported for other ENVs were projected on the three-dimensional EV11 structure (Figure 5). The possible relationship between them was detailed; however, we can only speculate about whether EV13 neutralization sites were affected or not. Two changes, A100V and F238I, were structurally close to known neutralization site I described for poliovirus (*13*), CBV4 (*32*), and CBV5 and SVDV (*14*). However, in most cases the amino acid differences were located in the VP1-2A junction (S274A, T276P, N284S, P285T, A286G, S287G, K288R, M290V, N291T, and H292N), affecting a region that was considered part of the neutralizing antigenic site III for SVDV (*15*). The amino acid changes (S274A and T276P) implied a change in the properties from hydrophilic to hydrophobic residues. These two positions were also different from the reference strain in the two U.S. isolates; however, they did not result in amino acids with hydrophobic characteristics (T276D/E).

Apart from the American isolates, the comparison of the amino acid sequence for EV13 isolates with respect to the reference strain was only possible in the amino terminal part of the VP1 protein. In this region, changes were identified in the strains that dated from 1965 to 1979, mostly affecting amino acids that have been considered as part of the neutralizing antigenic site I in poliovirus (D84S and A85D/E) (*13*). Only one amino acid (M216I) differed between the two Spanish clusters. This position was not related to any known antigenic site.

## Discussion

After a 12-years period in which EV13 circulation in Spain was not detected, a virus identified through neutralization test as EV13 began to be isolated in CSF of patients with aseptic meningitis. These isolates were also clearly identified as EV13 serotypes through molecular typing by their substantial homology with the EV13 Del Carmen prototype strain. The viruses could also be identified directly from most of the CSF samples assayed (three of them included in this study; other data not shown) without the need of isolation, demonstrating the utility and reliability of the ENV molecular typing according to the VP1 homology with respect to the reference strains (*11*). Indeed, the molecular study was successfully completed, despite the high degree of genetic differences between the EV13 recent isolates and the prototype strains of EV13 and ENV69, which were isolated in the Philippine Islands in 1953 and in Mexico before 1973, respectively.

Phylogenetic analysis of the nucleotide sequences showed that the Spanish strains were clustered into two distinct groups, those from the Canary Islands and those from the Iberian Peninsula, coinciding with their geographic origin. The first aseptic meningitis case caused by EV13 was described in the Canary Islands in February 2000. The distinctness of the groups was indicated by several silent nucleotide changes and one amino acid change, which apparently developed in a short period (the initial isolate of both groups was obtained about 1 month apart); for this reason, we cannot rule out the possibility that both groups could have had a common form but different ancestor. The special climate of the Canary Islands could explain the winter appearance of the cases (first case February 14).

When both Spanish clusters were genetically compared with isolates from other geographic origins, the EV13 strains from the Canary Islands appeared more closely related to the German isolates of 2000. This may reflect the introduction of EV13 from the Canary Islands into northern Europe (or vice versa) through tourist travel. The European isolates (Swedish, Italian, and German) obtained before 2000 presented nucleotide distances in respect to the Spanish isolates that increased over time. On the other hand, the two American strains, obtained in 1986 and 1995, showed the most distance between isolates. Indeed, the Spanish isolates showed larger differences with respect to the American isolates than those identified with the rest of the available EV13 sequences. In addition, the American strains of EV13 seem to be unrelated to the EV13 strains reported in the rest of the world; these American strains may represent another evolutionary lineage of this serotype. As a result, European and American EV13 strains could be assigned to different subgroups, in contrast to EV30 strains, which do not seem to be restricted geographically (*12*). However, more U.S. isolates from the same period would be necessary to define a consistent subgroup. Yet, preliminary data show that the reported U.S. isolates from 2001 may be genetically close to the European isolates (*33*).

The correlation between the genetic distance and the time parallels the behavior of such other epidemic viruses as EV30 (*12*), EV7 (*34*), EV25 (5), and ENV71 (*35*). However, these viruses usually maintain their isolation rates and increase their circulation periodically (*28*). In contrast, EV13 was isolated infrequently throughout the world until 2000, when isolation rates increased dramatically in several countries in association with aseptic meningitis outbreaks. This type of presentation was previously described in other HEVs such as EV4, which caused a large aseptic meningitis outbreak in 1997 in Israel (*36*). This behavior may be due to a genetic and antigenic drift that changes the virologic properties of the isolates. However, EV13 may simply be rare, and artifacts of surveillance or identification may have led to its seemingly sudden appearance. In consequence, viruses could have increased in virulence or improved their pathogenic mechanisms by antigenic change, despite the fact that they were able to be neutralized in vitro with horse-specific serum. However, further study is needed to investigate their antigenic characteristics, the human immune response to EV13, and whether a change in virulence occurs. The study of the VP1 genome region permitted us to observe the characteristics of the major capsid protein, which may be involved in this type of process, together with the rest of structural proteins. However, other genome changes, including those affecting replication (*37*,*38*), may also be implicated; thus, to understand the molecular epidemiology of HEV outbreaks, molecular studies of other regions of the genome are indicated.

Changes in EV13 coding sequence involved several differences in the C-terminal extreme of the VP1 protein, known to be highly variable and containing neutralizing antigenic site III in SVDV (*15*). Similar alterations have been related to positive selection affecting other ENV as EV30 (*12*), determining its evolution over the time. These amino acid changes in EV13 VP1 protein could be involved in the notable increase in the number of isolations of these viruses, since they were able to cause aseptic meningitis outbreaks worldwide (*19*–*23*,*25*,*27*,*39*). Moreover, although the isolates were successfully neutralized in vitro with the Lim-Benyesh-Melnick immune serum pools, the amino acid changes affecting VP1 should be taken into account during the evaluation of the neutralization test results. The only amino acid change between the two mentioned Spanish groups (M216I), which entails a change of a hydrophilic amino acid into a hydrophobic one, might confer some biologic advantage. It is not related to any neutralizing antigenic site previously described for ENVs, but might be related to the canyon structure, since this position is located in the floor of this structure in polioviruses (*40*). However, further research on the interaction between the cellular receptor and EV13 canyon should address this point. The canyon structure is a capsid surface depression described in several ENVs and rhinoviruses (*16*) that has been reported to join different cellular receptors (*41*). Recently, studies of the EV11 receptor (*31*) indicate that differences of few amino acid residues of the viral surface affecting the canyon structure may change the infectivity of the virus dramatically.

The molecular epidemiology of EV13 presents similarities and differences with other HEVs. The studies to date have demonstrated an epidemic pattern in viruses related with outbreaks such as ENV70 and EV30. This pattern suggests a unique strain of virus causing outbreaks all over the world similar the epidemics attributable to influenza A and B viruses.

However, since the human population is believed to be the only ENV reservoir, this pattern should be completed with the continuous isolation of the agent over nonepidemic years. This type of circulation has been observed in several HEVs, such as EV30 or EV9 but has not been observed in the case of EV13 or other rare HEVs. However, EV13 may have been circulating in the population at undetectable levels or in a nonpathogenic way. Since HEVs are usually typed only when they are related to unexpected outbreaks, a nonpathogenic virus could circulate in the population for a number of years before causing outbreaks.

The circulation of EV13 in Spain seem to have suddenly stopped. Since October 2000, over 700 ENV isolates have been typed in the Spanish Enterovirus Reference Laboratory, and only 1 imported EV13 isolate (data not shown) was detected in 2001 in a tourist from the Czech Republic who was visiting the Canary Islands.

## References

[1] Melnick JL. Enteroviruses: polioviruses, coxsackievirus, echoviruses and newer enteroviruses. In: Raven L, editor. Fields virology. Philadelphia: Lippincott-Raven; 1996. p. 655–712.

[2] Family *Picornaviridae*. In: Regenmortel M, editor. Virus taxonomy. Seventh report of the International Committee on Taxonomy on Viruses. San Diego: Academic Press; 2000.

[3] Poyry T, Kinnunen L, Hyypia T, Brown B, Horsnell C, Hovi T, Genetic and phylogenetic clustering of enteroviruses. J Gen Virol. 1996;77:1699–717. 10.1099/0022-1317-77-8-1699 8760417

[4] Kunkel U, Schreier E. Genetic variability within the VP1 coding region of echovirus type 30 isolates. Arch Virol. 2000;145:1455–64. 10.1007/s007050070102 10963349

[5] Kunkel U, Schreier E. Characterization of echovirus 25 (ECV 25) in the VP1/2A gene junction region. Brief report. Arch Virol. 1999;144:2253–8. 10.1007/s007050050640 10603180

[6] Mulders MN, Salminen M, Kalkkinen N, Hovi T. Molecular epidemiology of coxsackievirus B4 and disclosure of the correct VP1/2A(pro) cleavage site: evidence for high genomic diversity and long-term endemicity of distinct genotypes. J Gen Virol. 2000;81:803–12. 10675418 10.1099/0022-1317-81-3-803

[7] Oberste MS, Maher K, Kilpatrick DR, Flemister MR, Brown BA, Pallansch MA. Typing of human enteroviruses by partial sequencing of VP1. J Clin Microbiol. 1999;37:1288–93. 10203472 10.1128/jcm.37.5.1288-1293.1999 PMC84754

[8] Caro V, Guillot S, Delpeyroux F, Crainic R. Molecular strategy for “serotyping” of human enteroviruses. J Gen Virol. 2001;82:79–91. 11125161 10.1099/0022-1317-82-1-79

[9] Norder H, Bjerregaard L, Magnius LO. Homotypic echoviruses share aminoterminal VP1 sequence homology applicable for typing. J Med Virol. 2001;63:35–44. 10.1002/1096-9071(200101)63:1<35::AID-JMV1005>3.0.CO;2-Q 11130885

[10] Casas I, Palacios GF, Trallero G, Cisterna D, Freire MC, Tenorio A. Molecular characterization of human enteroviruses in clinical samples: comparison between VP2, VP1, and RNA polymerase regions using RT nested PCR assays and direct sequencing of products. J Med Virol. 2001;65:138–48. 10.1002/jmv.2013 11505456

[11] Palacios G, Casas I, Tenorio A, Freire C. Molecular identification of enterovirus by analyzing a partial VP1 genomic region with different methods. J Clin Microbiol. 2002;40:182–92. 10.1128/JCM.40.1.182-192.2002 11773114 PMC120085

[12] Palacios G, Casas I, Cisterna D, Trallero G, Tenorio A, Freire C. Molecular epidemiology of echovirus 30: temporal circulation and substitution of single lineages. J Virol. 2002;76:4940–9. 10.1128/JVI.76.10.4940-4949.2002 11967311 PMC136144

[13] Minor PD, Ferguson M, Evans DM, Almond JW, Icenogle JP. Antigenic structure of polioviruses of serotypes 1, 2 and 3. J Gen Virol. 1986;67:1283–91. 10.1099/0022-1317-67-7-1283 2425046

[14] Borrego B, Carra E, Garcia-Ranea JA, Brocchi E. Characterization of neutralization sites on the circulating variant of swine vesicular disease virus (SVDV): a new site is shared by SVDV and the related coxsackie B5 virus. J Gen Virol. 2002;83:35–44. 11752698 10.1099/0022-1317-83-1-35

[15] Nijhar SK, Mackay DK, Brocchi E, Ferris NP, Kitching RP, Knowles NJ. Identification of neutralizing epitopes on a European strain of swine vesicular disease virus. J Gen Virol. 1999;80:277–82. 10073685 10.1099/0022-1317-80-2-277

[16] Rossmann MG. The canyon hypothesis. Hiding the host cell receptor attachment site on a viral surface from immune surveillance. J Biol Chem. 1989;264:14587–90. 2670920

[17] Savolainen C, Hovi T, Mulders MN. Molecular epidemiology of echovirus 30 in Europe: succession of dominant sublineages within a single major genotype. Arch Virol. 2001;146:521–37. 10.1007/s007050170160 11338388

[18] Oberste MS, Maher K, Kennett ML, Campbell JJ, Carpenter MS, Schnurr D, Molecular epidemiology and genetic diversity of echovirus type 30 (E30): genotypes correlate with temporal dynamics of E30 isolation. J Clin Microbiol. 1999;37:3928–33. 10565909 10.1128/jcm.37.12.3928-3933.1999 PMC85848

[19] Echovirus type 13—United States, 2001. MMWR Morb Mortal Wkly Rep. 2001;50:777–80. 11570484

[20] Cunney R. Enteroviral infections: a common cause of viral meningitis. Epi-Insight. 2001;2:2–3.

[21] Recent increases in incidence of echoviruses 13 and 30 around Europe. Eurosurveillance Weekly. 2002;14:7.

[22] Diedrich S, Schreier E. Aseptic meningitis in Germany associated with echovirus type 13. Biomed Center Infectious Diseases. 2001;1:14. 10.1186/1471-2334-1-14 11591222 PMC57743

[23] Viral meningitis associated with increase in echovirus type 13. Communicable Disease Report–Weekly 2000;10:277,280. 10948792

[24] Noah N. Recent increases in incidence of echovirus 13 and 30 around Europe. Eurosurveillence Weekly 2002;Feb 14:7. Available from: URL: http://www.eurosurv.org/update/

[25] Trallero G, Casas I, Avellón A, Pérez C, Tenorio A, de la Loma A. First epidemic of aseptic meningitis due to echovirus type 13 among Spanish children. Epidemiol Infect. 2003;130:251–6. 10.1017/S0950268802008191 12729193 PMC2869960

[26] Keino M, Kanno M, Hirasawa K, Watari T, Mikawa M, Saito K, Isolation of echovirus type 13 from patients of aseptic meningitis. Jpn J Infect Dis. 2001;54:249–50. 11862012

[27] McMinn P. Echovirus meningitis in Western Australia. Communicable Diseases—Australia 2001; September.

[28] Trallero G, Casas I, Tenorio A, Echevarria JE, Castellanos A, Lozano A, Enteroviruses in Spain: virological and epidemiological studies over 10 years (1988–97). Epidemiol Infect. 2000;124:497–506. 10.1017/S0950268899003726 10982074 PMC2810936

[29] Casas I, Powell L, Klapper PE, Cleator GM. New method for the extraction of viral RNA and DNA from cerebrospinal fluid for use in the polymerase chain reaction assay. J Virol Methods. 1995;53:25–36. 10.1016/0166-0934(94)00173-E 7635925

[30] Oberste MS, Maher K, Flemister MR, Marchetti G, Kilpatrick DR, Pallansch MA. Comparison of classic and molecular approaches for the identification of untypeable enteroviruses. J Clin Microbiol. 2000;38:1170–4. 10699015 10.1128/jcm.38.3.1170-1174.2000 PMC86366

[31] Stuart AD, McKee TA, Williams PA, Harley C, Shen S, Stuart DI, Determination of the structure of a decay accelerating factor-binding clinical isolate of echovirus 11 allows mapping of mutants with altered receptor requirements for infection. J Virol. 2002;76:7694–704. 10.1128/JVI.76.15.7694-7704.2002 12097583 PMC136386

[32] Halim S, Ramsingh AI. A point mutation in VP1 of coxsackievirus B4 alters antigenicity. Virology. 2000;269:86–94. 10.1006/viro.2000.0188 10725201

[33] Mullins A. Changes in circulating enterovirus serotypes in the United States, 2001. In International Conference on Emerging Infectious Diseases, Atlanta, Georgia, USA, March 22–24, 2002. Slide session available from: URL: http://www.cdc.gov/iceid/webcast/latebreakers1.htm

[34] Chua BH, McMinn PC, Lam SK, Chua KB. Comparison of the complete nucleotide sequences of echovirus 7 strain UMMC and the prototype (Wallace) strain demonstrates significant genetic drift over time. J Gen Virol. 2001;82:2629–39. 11602774 10.1099/0022-1317-82-11-2629

[35] Chu PY, Lin KH, Hwang KP, Chou LC, Wang CF, Shih SR, Molecular epidemiology of enterovirus 71 in Taiwan. Arch Virol. 2001;146:589–600. 10.1007/s007050170164 11338392

[36] Handsher R, Shulman LM, Abramovitz B, Silberstein I, Neuman M, Tepperberg-Oikawa M, A new variant of echovirus 4 associated with a large outbreak of aseptic meningitis. J Clin Virol. 1999;13:29–36. 10.1016/S1386-6532(99)00014-1 10405889

[37] Oprisan G, Combiescu M, Guillot S, Caro V, Combiescu A, Delpeyroux F, Natural genetic recombination between co-circulating heterotypic enteroviruses. J Gen Virol. 2002;83:2193–200. 12185273 10.1099/0022-1317-83-9-2193

[38] Norder H, Bjerregaard L, Magnius LO. Open reading frame sequence of an Asian enterovirus 73 strain reveals that the prototype from California is recombinant. J Gen Virol. 2002;83:1721–8. 12075091 10.1099/0022-1317-83-7-1721

[39] Isolation of echovirus 13 from meningitis cases—September 2001 Fukushima. Infectious Agent Surveillance Report 2001;22(12 December).

[40] He Y, Bowman VD, Mueller S, Bator CM, Bella J, Peng X, Interaction of the poliovirus receptor with poliovirus. Proc Natl Acad Sci U S A. 2000;97:79–84. 10.1073/pnas.97.1.79 10618374 PMC26619

[41] Rossmann MG, He Y, Kuhn RJ. Picornavirus-receptor interactions. Trends Microbiol. 2002;10:324–31. 10.1016/S0966-842X(02)02383-1 12110211

